# Endurance, Refuge, and Reemergence of Dengue Virus Type 2, Puerto Rico, 1986–2007

**DOI:** 10.3201/eid1701.100961

**Published:** 2011-01

**Authors:** Kate L. McElroy, Gilberto A. Santiago, Niall J. Lennon, Bruce W. Birren, Matthew R. Henn, Jorge L. Muñoz-Jordán

**Affiliations:** Author affiliations: Centers for Disease Control and Prevention, San Juan, Puerto Rico (K.L. McElroy, G.A. Santiago, J.L. Muñoz-Jordán);; Broad Institute of MIT and Harvard, Cambridge, Massachusetts, USA (N.J. Lennon, B.W. Birren, M.R. Henn)

## Abstract

To study the evolution of dengue virus (DENV) serotype 2 in Puerto Rico, we examined the genetic composition and diversity of 160 DENV-2 genomes obtained through 22 consecutive years of sampling. A clade replacement took place in 1994–1997 during a period of high incidence of autochthonous DENV-2 and frequent, short-lived reintroductions of foreign DENV-2. This unique clade replacement was complete just before DENV-3 emerged. By temporally and geographically defining DENV-2 lineages, we describe a refuge of this virus through 4 years of low genome diversity. Our analyses may explain the long-term endurance of DENV-2 despite great epidemiologic changes in disease incidence and serotype distribution.

Epidemic dengue fever (DF) and the emergence of dengue hemorrhagic fever (DHF) in the Americas are associated with increased endemicity and cocirculation of the 4 dengue virus (DENV) serotypes, 1–4 (*1*). These increases have been particularly evident in Puerto Rico, where transmission increased during the past 25 years (*2**–**4*). The first DHF epidemics in the Americas occurred in the 1980s and were caused by the Asian/American genotype of DENV-2, then new to the region, which rapidly replaced the American genotype (*5**–**7*). This replacement has been linked to a potential to cause higher viremia and severe illness (*8**–**10*). Introduction of DENV-3 in the mid 1990s and increased human population and travel further fostered larger and more frequent DF and DHF epidemics in the region (*11**–**13*).

Although all 4 DENV serotypes circulate on the island, DENV-2 circulated continuously for 25 years. Previously, a partial sequence analysis from 74 DENV-2 isolates collected in Puerto Rico during 7 years throughout a 14-year period (1987–2001) showed a DENV-2 lineage evolving through a series of turnover events (*14*). A lineage replacement in 1994 appeared to be associated with a foreign virus but only 3 other reintroductions were found, all linked to the 1998 epidemic, the largest in Puerto Rico history (*14*). This was a turning point in the epidemiology of dengue, with DENV-2 (and DENV-1 and -4) rapidly declining during the expansion of DENV-3. However, transmission of DENV-2 persisted at low levels during 1999–2003 and increased thereafter. This serotype turnover offers new opportunities to study the evolution of DENV-2. Our analysis illustrates the genetic composition and population diversity of DENV-2 throughout 22 consecutive years of sampling in Puerto Rico and may explain the evolutionary resilience and long-term establishment of this virus.

## Methods

### Virus Isolates

We complied with the institutional review boards of the Centers for Disease Control and Prevention (CDC) (protocol 4797) and the Broad Institute of MIT and Harvard. DENV was obtained from human serum received through the passive surveillance system administered by CDC. Each sample was accompanied by a form that captured geographic and clinical information maintained for this study without patient identifiers. Primary or secondary status of infection was inferred by absence or presence of serum immunoglobulin G (*15*). Viruses were rescued into C6/36 cells (*16*). Selection of 3 isolates per year in the 5 municipalities with the highest reporting of DENV-2 cases resulted in 253 isolates, of which 140 were successfully sequenced and are representative of our virus repository with respect to patient age (27.7 vs. 22.6 years), sex (54.4% vs. 47.4% male), and history of infection (84.6% vs. 77% secondary infections). We also sequenced 20 regional isolates from neighboring countries.

### Sequencing

We extracted RNA from tissue culture supernatant using the M48 or MDx BioRobot (QIAGEN, Valencia, CA, USA). cDNA was generated by using Sensiscript RT (QIAGEN) with random hexamers (Applied Biosciences, Foster City, CA, USA). Presence of cDNA was confirmed by PCR by using *Pfu*UltraII (Stratagene, La Jolla, CA, USA) or iTaq (Bio-Rad, Hercules, CA, USA) DNA polymerase and specific oligonucleotides (CDC, Atlanta, GA, USA). Fourteen pooled overlapping 2,000 nt amplicons were generated by reverse transcription–PCR at CDC (San Juan, PR) and sequenced at the Broad Institute (Cambridge, MA, USA) by bidirectional Sanger by using an ABI 3730 after PCR with 96 M13-tailed serotype-specific primers. Resulting reads were trimmed of the primer sequences, filtered for high quality, and assembled by using algorithms developed by the Broad Institute. All coding sequences for the poliproteins (10,173 nt) and parts of the 5′ and 3′ noncoding regions were deposited in GenBank.

### Sequence Analyses

Coding sequences for the unprocessed polyprotein (5′ and 3′ noncoding regions excluded) were aligned by ClustalW software (www.ebi.ac.uk/Tools/clustalw/index.html) in MEGA 4 (www.megasoftware.net). Maximum likelihood analysis and bootstrapping tests were performed in PAUP* (*16*) under the best-fit substitution model estimated by MODELTEST v3.07 (*14*) (parameters available on request). The 1983 Jamaican isolate JM_83_M20558 (*5*) served as outgroup. Mean rates of nucleotide substitution and relative genetic diversity (*N*e*t*, where *t* is the generation time) were estimated by using Bayesian Markov Chain Monte Carlo (MCMC) from BEAST v1.4.7 (http://mbe.oxfordjournals.org/content/25/7/1459). General time reversible substitution model with strict and relaxed molecular clocks and constant population size or Bayesian Skyline coalescent analysis was used. All MCMC chains were run for sufficient length ensuring stationary parameters, with statistical error reflected in values of the 95% highest probability density. Amino acid differences were mapped by using parsimony methods in MacClade v4.08 (*17*). We determined d_N_/d_S_ ratios with the single likelihood ancestor counting method using HYPHY and accessed through the Datamonkey server (*13*). Associations between phylogeny and geographic data were investigated by using Bayesian Tip-association Significance testing (http://evolve.zoo.ox.ac.uk/evolve/BaTS.html) with the posterior sample of trees calculated by BEAST. For the parsimony score, association index, and monophyletic clade size, we considered p<0.05 significant.

## Results

**Figure 1:**
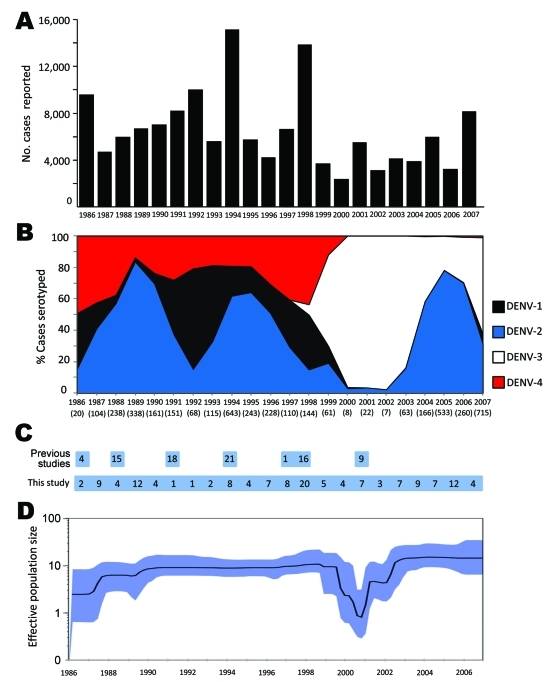
Historic overview of dengue, Puerto Rico, 1986–2007. A) Number of suspected, clinically defined cases of dengue fever/dengue hemorrhagic fever by year reported to the Centers for Disease Control and Prevention’s Dengue Branch. B) Percentage of identifications of each serotype relative to the total of positive serotype identifications by using tissue culture isolation or reverse transcription–PCR per year. Numbers in parenthesis indicate numbers of dengue virus (DENV) serotype 2 identifications each year. Black, DENV-1; blue, DENV-2; white, DENV-3; red, DENV-4. C) Number of partially sequenced (E gene) autochthonous Puerto Rican isolates reported by previous studies (*14*,*20*) and whole genome sequences obtained in the present study by year of their corresponding case presentation. D) Bayesian coalescent inference of population dynamics and genetic diversity by using the Bayesian Skyline plot. Markov Chain Monte Carlo from BEAST version 1.4.7 (www.biomedcentral.com/1741-7007/8/114). Sampling procedures were used to estimate posterior distribution of DENV-2 genetic diversity in an effective population through the study period on the basis of full genome sequence data. x axis, time in years through the study period; y axis, product of the effective population size (relative genetic diversity) and generation length in years; black line, median estimate; blue shadow, 95% highest probability density.

During 1986–2007, dengue cases in Puerto Rico ranged from 2,000 to ≈16,000 per year (Figure 1, panel A), with major epidemics (>8,000 cases) reported in 1986, 1992, 1994, 1998, and 2007 (*2**–**4**,**18**,**19*). Despite major fluctuations in serotype circulation, DENV-2 circulated predominantly for 10 years (Figure 1, panel B), alternating with DENV-1 through 2 periods of resurgence during the 1990s and cocirculation of DENV-4 (Figure 1, panel B). DENV-2 declined markedly after the 1998 epidemic and the dissemination of DENV-3 concomitant to the disappearance of DENV-1 and -4. However, DENV-2 continued to cause a low number of cases during 1999–2003 and reemerged in 2004–2007 (Figure 1, panel B). Samples from every year of the 22-year study period (Figure 1, panel C) comprised our analysis.

**Figure 2:**
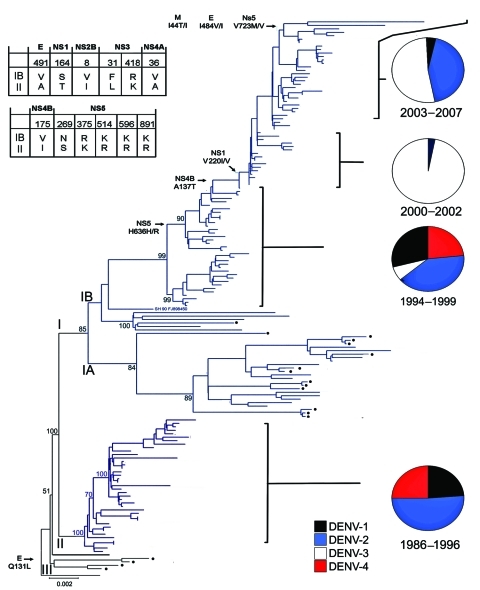
Evolution of dengue virus (DENV) serotype 2, Puerto Rico. Maximum likelihood phylogeny of the 140 Puerto Rico and 20 international isolates of DENV-2 (see number of isolates by year below). Names of clades (I, II, and III) and subclades (IA, IB) are shown at the base of their respective branches on the phylogeny tree. Clade II (dark blue) circulated during 1986–1996 and clade I (light blue) during 1994–2007. Subclade IA and clade III represent foreign, transient reintroductions throughout the 22-year study period. Black dots indicate 18 isolates from Puerto Rico with phylogenetic associations closer to foreign isolates than to other Puerto Rico viruses. Two-letter geographic codes indicate origin (PR, Puerto Rico; JM, Jamaica; SJ, Saint John; SH, Saint Thomas; VE, Venezuela; Cl, Colombia; NC, Nicaragua; MX, Mexico; CU, Cuba; SI, Saint Kits; DR, Dominican Republic; SX, Saint Croix; BR, Brazil). PR code is accompanied by a 2-digit number that indicates geographic location (municipality) on the island. The 2-digit numbers between the geographic codes and the GenBank accession numbers indicate the year of the corresponding case of each isolate. Bootstrap values are shown for all clades (I, II, and II), subclades (IA, IB), and most immediate lineages. Twelve amino acid changes associated with IB/II differences are shown on the Table; 6 other selected changes across subclade IB are shown on the left at the base of the branches exhibiting the respective changes. Amino acid numbers refer to the position in each DENV protein. Relevant epidemiologic events are highlighted to the right with pie charts showing the relative levels of each serotype isolated for that period Black, DENV-1; blue, DENV-2; white, DENV-3; red, DENV-4. Scale bar indicates nucleotide substitutions per site.

**Figure A1:**
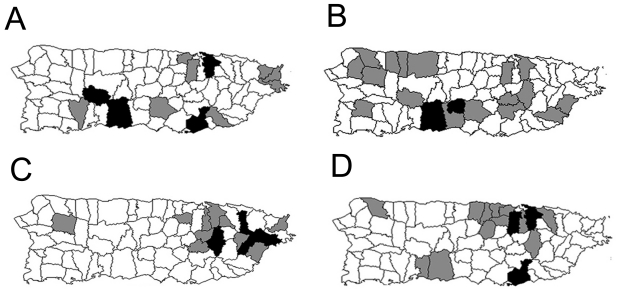
Dengue virus (DENV) serotype 2 case distribution, Puerto Rico, 1987–2006. Maps of Puerto Rico are shown as examples of distribution of total of DENV-2 cases reported on the island during 4 different years representing clade II expansion (1987) (A), clades II/I mixing (1995) (B), low evolution of subclade IB (2001) (C), and resurgence of subclade IB (D). All maps represent the 78 municipalities of Puerto Rico. White, 0–1 DENV-2 identifications; gray, 2–5 DENV-2 identifications; black, >6 DENV-2 identifications. Maximum number of identifications in any 1 municipality was 17.

The Bayesian Skyline analysis (Figure 1, panel D) of the autochthonous viral sequences (Figure 2; clades IB and II Figure A1) showed a gradual increase in the genetic diversity of DENV-2 during 1987–1991 that corresponds to a period of high transmission and dominance (Figure 1, panel B). This increase was followed by 9 years of high genetic diversity that coincided with a period of DENV-1 and DENV-4 cocirculation. The genetic diversity of DENV-2 declined sharply during 1999–2003, coinciding with a period of minimal DENV-2 transmission. Genetic diversity rebounded in 2005 to roughly pre-1999 levels as the virus reemerged.

The densely populated island of Puerto Rico (3,808,610 population; 3,508 square miles) is divided into 78 municipalities grouped in 8 regions. The 140 DENV-2 genomes from 37 municipalities represented all 8 regions and ranged from 2–20 isolates per year (Figure 1, panel C; Figure 2). We included 20 other sequences from Caribbean countries. The number of Puerto Rico sequences is proportional to the epidemic level or the relative proportion of DENV-2 identifications in the municipalities with highest DENV-2 reporting per year. The phylogeny of the 160 DENV-2 genomes showed 2 major clades (I and II) and a smaller clade (III) (Figure 2). Clade I contains 115 sequences and can be further subdivided in 2 subclades. Subclade IA (1998–2007) contains 13 Puerto Rico and 12 Caribbean sequences, including 1 from St. Thomas (SH 90 FJ898450) identified as the closest ancestor. Subclade IB (1994–2007) contains 90 sequences mostly of local origin, but the presence of 6 foreign and 1 local basal sequences confirms its foreign origin. Clade II (1986–1994) contains 39 local isolates. Basal to clades I and II is a St. John 1987 sequence (SJ 87 GQ868603), which suggests a possible origin. Clade III is formed by 4 local isolates during1987–1991. These genetically distinct isolates do not fit in clade I or II, but a separate analysis with publically available envelope gene sequences pointed to possible Caribbean origin (K.L. McElroy et al., unpub. data).

Four events merit recognition (Figure 2). First, a mixture of foreign and local strains at the base of subclades IA and IB provides evidence of multiple introductions. Eight Puerto Rico viruses associated with these foreign strains date from 1994 through 1999. These years also are associated with a distinct subgroup basal to subclade IB concomitant with the extinction of clade II in 1997. Second, subclade IB evolved mainly after the introduction of DENV-3 in 1998. Third, a period of limited circulation of DENV-2 reflected in low levels of genetic diversity (1999–2003) coincided with the expansion of DENV-3 and decline of DENV-1 and -4. Fourth, there was a resurgence of DENV-2 during 2004–2007.

Forty-nine amino acid differences mapped to the phylogeny were detected across the major internal branches of the tree. Twenty of these comprise major differences between clades I and II and between subclades IA and IB, as well as substitutions that arose during the continuous evolution of subclade IB (Figure 2). Only 1 aa substitution distinguished isolates in clades I/II from III: a hydrophilic glutamine to a hydrophobic leucine at position 131 in the E protein. Excluding PR79_1995_EU569708 as a possible foreign introduction, 18 aa differences distinguish isolates across clade I, 12 of which separate subclade IB from clade II and potentially could have been involved in the 1994–1997 lineage turnover (Figure 2). The remaining differences between isolates in subclades IB and II were present in nonstructural (NS) genes and are preponderantly conservative mutations, with the exception of position 31 in NS3, which was nonconservative. Among the additional changes, the only nonconservative mutation was a hydrophobic alanine to hydrophilic threonine at position 137 in NS4B that originated with PR40_1999 EU482730, and most changes were found in the NS genes.

Using Bayesian MCMC and d_N_/d_S_ analyses, we estimated the mean substitution rates for the full genomes at 9 × 10^–4^ to 1.1 × 10^–3^ for all clades, consistent with previously published rates (*20**,**21*). The low d_N_/d_S_ ratios (0.07–0.08) provide evidence of a low percentage of substitutions that have been fixed along independent lineages, possibly indicating purifying, negative selection.

**Figure 3:**
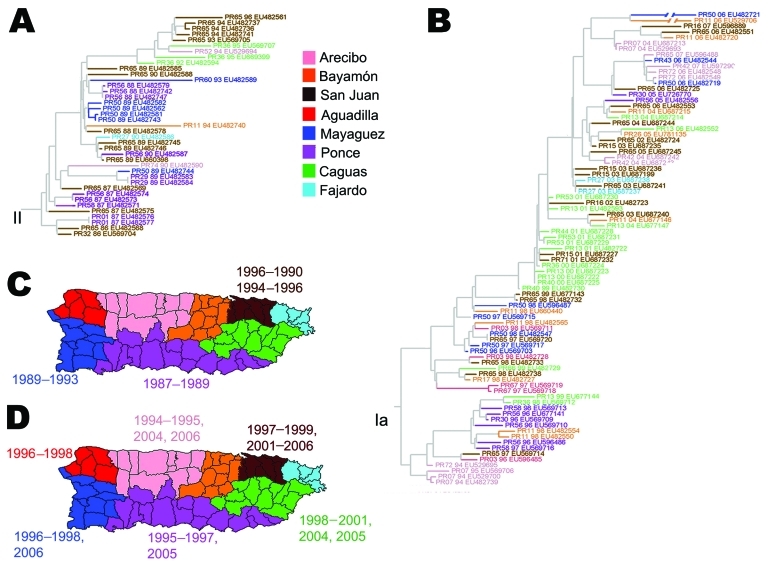
Geographic clustering of Puerto Rico dengue virus lineages. A) Maximum-likelihood phylogeny of clade II. All isolates indicate year of case presentation and GenBank accession numbers. B) Maximum-likelihood phylogeny of subclade IB shows isolates by year and GenBank accession numbers. Six regions had >5 isolates (San Juan, Caguas, Ponce, Mayaguez, Aguadilla, and Arecibo). C) Eight regions of Puerto Rico with colors corresponding to isolates in panel A and year for the 3 regions with more isolates of that clade: San Juan, Mayaguez, and Ponce. D) Eight regions of Puerto Rico, showing colors and years corresponding to isolates in panel B. Correlation between phylogeny and geographic location of isolation for the isolates in this study was estimated by using Bayesian Tip-association Significance testing. Association index 6.51 (95% confidence interval 6.03–7.22); parsimony score statistic 52.27 (95% confidence interval 51–54). Monophyletic clade size Bayesian Tip-association Significance estimates are shown for 6 regions of Puerto Rico with >5 isolates represented in at least 1 subclade and statistically representative geographic associations (p<0.05).

BaTS analysis shows that lineages often correlated with the corresponding region of origin of the isolates. Seven of the 8 regions had >4 isolates in subclade IB or clade II. This association was significant for 6 regions (p<0.05) (Table). The most significant geographic correlation of lineages were found in the San Juan (1986–1990 and 1994–1996), Ponce (1987–1989), and Mayaguez (1989 and 1993) (Figure 3, panel A). In addition, isolates clustered geographically for San Juan (1997–1999 and 2001–2006), Caguas (1998–2001, 2004, and 2005), Ponce (1995–1997 and 2005), Mayaguez (1996–1998 and 2006), Aguadilla (1996–1998), and Arecibo (1994–1995, 2004, and 2006) (Figure 3, panel B ). Considering the DENV-2 historical data, we recognize that high-reporting municipalities usually are located in regions where we identified significant phylogenetic clustering (Figure A1). For example, in 1987, most DENV-2 cases originated from the Ponce and San Juan regions, where we identified lineages of clade I. For1994–1996, DENV-2 cases in San Juan, Ponce, Mayaguez, and Arecibo regions may reflect the coexistance of subclades IB and clade II.

**Table:** Correlation between phylogeny and geographic location of dengue virus isolation, Puerto Rico 1986–2007*

Clade	Region	No. isolates	Estimated BaTs (95% HPD CIs)	p value
I B	Aguadilla	5	2 (2–2)	0.04
	Arecibo	12	3 (3–3)	0.03
	Caguas	17	2.75 (2–3)	0.04
II	Mayaguez	6	4 (4–4)	0.01
	Ponce	11	3 (3–3)	0.05
	San Juan	15	5 (5–5)	0.01

*Correlation estimated by using BaTs. Association index = 6.51 (95% CI 6.03–7.22). Parsimony score statistic = 52.27 (95% CI 51–54). BaTs, Bayesian Tip-association Significance testing; HPD, highest probability density; CI, confidence interval.

We investigated other possible associations with the DENV-2 phylogeny, including age and DF/DHF status, but found none. Most DENV-2 infections were secondary (84.6% and 77% of DENV-2 infections in the CDC collection and this study, respectively). However, we found no relationship between phylogeny and incidence of primary or secondary infection in patients.

**Figure 4:**
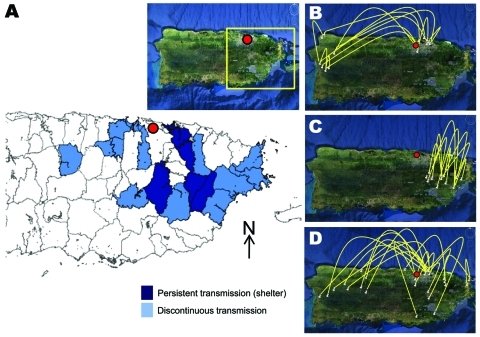
Epidemiology of dengue virus (DENV) serotype 2 in Puerto Rico, 1997–2006. A) Municipalities with persistent DENV-2 transmission (Caguas, Juncos, Las Piedras, Carolina) versus those with discontinuous transmission (Morovis, Toa Alta, Toa Baja, Cataño, Guaynabo, Cidra, San Lorenzo, Canóvanas, Humacao, Naguabo, Ceiba, Fajardo), 1998–2002. Inset shows satellite view; red dot indicates national capital (San Juan), and yellow box indicates region where DENV-2 took refuge during 2000–2002. B–D) Satellite view depicts virus transmission corridors. White pins point to specific geographic locations where DENV-2 isolates were collected during the specified time period. Yellow lines connect isolates by their phylogenetic affiliations suggesting migration of virus. B) DENV-2 traveled to the San Juan region from the west during 1997–1999; C) DENV-2 transmission retracted to the eastern, refuge region with restricted dispersion patterns during 2000–2002; D) DENV-2 reemerged focused on the San Juan region and later dispersed throughout the island during 2003–2006.

**Figure 5:**
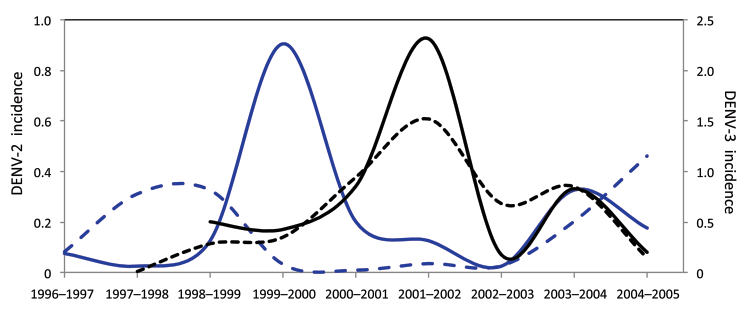
Incidence of dengue virus (DENV) serotypes 2 and 3 in Puerto Rico, 1996–2005. Solid blue line, incidence of DENV-2 within the refuge region; dashed blue line, incidence of DENV-2 in the rest of the island outside the refuge reason; solid black line, incidence of DENV-3 within the DENV-2 refuge region; dashed black line) incidence of DENV-3 in the rest of the island outside the refuge region. Incidence was calculated as number of confirmed, positive cases of each serotype per thousand residents.

The year 1999 began a period of low circulation and low genetic diversity of the Caguas lineage of subclade IB (Figure 1, panel D; Figure 2; Figure 3, panel B) that lasted until 2003. During these 4 years, most DENV-2 cases originated from only 4 municipalities in eastern Puerto Rico (Figure 4, panel A); <20 additional DENV-2 cases were reported during that period in 12 other neighboring municipalities (Figure 4, panel A). Because phylogenetic lineages are geographically and temporally clustered, (Figure 2), we illustrated these associations on the map of Puerto Rico (Figure 4). This map shows that DENV-2 descendants from western Puerto Rico emerged in San Juan in 1997–1998 (Figure 4, panel B, top), then appeared and persisted within the refuge in 1999–2002 (Figure 4, panel C, middle) to then disseminate across the island in 2003–2005 (Figure 4, panel D). In the 4 municipalities with uninterrupted DENV-2 transmission, DENV-2 incidence increased 2 years after the islandwide increase (Figure 5). DENV-3 incidence within this DENV-2 refuge was minimal during the period of high DENV-2 incidence but peaked 2 years later concomitant with an increase across the rest of the island

## Discussion

Puerto Rico is a model for fine-scale studies on DENV evolution in the Americas. The long-term persistence of DENV-2 and its ability to reemerge after transient periods of low circulation is a remarkable aspect of the epidemiology of dengue in the region. The fact that 13% of DENV-2 isolates represent importations or close descendants from importations brings new insights to our understanding of DENV long-term circulation. Foreign viruses were identified in 8 years (1987, 1989, 1991, 1995, 1998, 1999, 2005, and 2007), of which only 1991 and 1998 had been previously sampled (*14*). Ten of the 18 introductions occurred during periods of high DENV-2 predominance: 1987–1991, 1995, and 2005–2007 (Figure 1, panel B; Figure 2). The other 8 introductions originated from the 1998 epidemic or shortly thereafter (1999). Therefore, DENV-2 seems to be introduced mainly during periods of favorable preponderance, not necessarily epidemic transmission of this serotype. Subclade IA viruses never established themselves, regardless of year of isolation or origin. These assessments showed a previously unknown feature of DENV-2 persistence: the endemic strain is recalcitrant to influences from frequent foreign introductions.

The relative inability of “foreign” DENV-2 to persist in the presence of the dominant subclade IB viruses is not well understood. The Puerto Rico strain might be highly adapted and thus have a fitness advantage, the frequently introduced strains might be simply underrepresented, or introduced strains may have disappeared through genetic drift. Isolate PR76_1995_EU569708, which lies basal to this subclade in the phylogeny (Figure 2), is more closely related to South American DENV-2 viruses than to other Puerto Rico viruses, and this lineage does not appear to have progressed, supporting the foreign origin of subclade IB. Our findings then show that subclade IB resulted from an introduced strain, as previously suggested by Bennett et al. (*14*), and successfully penetrated during a period of proportionally high incidence of foreign introductions. Interestingly, this clade replacement was completed in 1997, less than a year before the finding of DENV-3 and the concomitant decline of DENV-1, -2, and -4. The early portion of subclade IB is seen as a period of short-lived lineages ending in 1997, therefore, the rise and expansion of this subclade mainly occurs in coexistence with DENV-3, a different epidemiologic scenario from that of the now extinct clade II a decade earlier.

The dominance of conservative amino acid changes that segregated the viruses by clade hinders the assessment of phenotypic changes. Compensatory mutations might have conferred replicative advantages that could have influenced the displacement of clade II or the persistence of subclade IB in Puerto Rico; however this hypothesis has not been tested. Positive selection was not identified, contrasting with previous analyses (*14**,**22**–**24*). Others have not detected positive selection and attribute lineage extinctions or clade replacements to stochastic events rather than natural selection (*25*). More analysis to detect site-specific selection is needed to corroborate whether positive selection is not at play in these populations of viruses.

The period 1999–2003 represents historically low rates of DENV-2 circulation (Figures 1, 2, 4), and the epidemiologic and phylogenetic aspects of this transient retrieval had not been studied previously. We show that the genetic variability of DENV-2 decreased during these 4 years when the virus was transmitted in only a subset of municipalities. DENV-2 represented 29% of the cases in this area but only 5% island-wide. The reason this region became a refuge of DENV-2 for 4 years remains unclear, but the low incidence of DENV-2 in prior years compared with the rest of the island suggests susceptibility for infection in this population (Figure 5). Studies in Thailand showed serotype displacement affecting population diversity and lineage turnover (*26*). Short-term serotype cross-protection has been suggested to contribute to serotype displacements (*27**–**29*), implying that as DENV-3 infected a large susceptible population, cross-protective antibodies momentarily impeded transmission of other serotypes and dissemination of DENV-2 outside the eastern refuge. Our study confirms the utility of systematic sampling and genome sequencing in large-scale surveillance systems as ways to understand the dynamics of dengue transmission and endemicity.
